# Porous Nanostructure, Lipid Composition, and Degree of Drug Supersaturation Modulate In Vitro Fenofibrate Solubilization in Silica-Lipid Hybrids

**DOI:** 10.3390/pharmaceutics12070687

**Published:** 2020-07-21

**Authors:** Ruba Almasri, Paul Joyce, Hayley B. Schultz, Nicky Thomas, Kristen E. Bremmell, Clive A. Prestidge

**Affiliations:** 1 UniSA Clinical and Health Sciences, University of South Australia, Adelaide 5000, Australia; Ruba.Almasri@mymail.unisa.edu.au (R.A.); Paul.Joyce@unisa.edu.au (P.J.); Hayley.Schultz@unisa.edu.au (H.B.S.); Nicky.Thomas@unisa.edu.au (N.T.); Kristen.Bremmell@unisa.edu.au (K.E.B.); 2 ARC Centre of Excellence in Convergent Bio-Nano Science and Technology, University of South Australia, Adelaide 5000, Australia

**Keywords:** supersaturation, silica-lipid hybrid, spray drying, lipolysis, lipid-based formulation, fenofibrate, mesoporous silica, oral drug delivery

## Abstract

The unique nanostructured matrix obtained by silica-lipid hybrids (SLHs) is well known to improve the dissolution, absorption, and bioavailability of poorly water-soluble drugs (PWSDs). The aim of this study was to investigate the impact of: (i) drug load: 3–22.7% *w/w*, (ii) lipid type: medium-chain triglyceride (Captex 300) and mono and diester of caprylic acid (Capmul PG8), and (iii) silica nanostructure: spray dried fumed silica (FS) and mesoporous silica (MPS), on the in vitro dissolution, solubilization, and solid-state stability of the model drug fenofibrate (FEN). Greater FEN crystallinity was detected at higher drug loads and within the MPS formulations. Furthermore, an increased rate and extent of dissolution was achieved by FS formulations when compared to crystalline FEN (5–10-fold), a commercial product; APO-fenofibrate (2.4–4-fold) and corresponding MPS formulations (2–4-fold). Precipitation of FEN during in vitro lipolysis restricted data interpretation, however a synergistic effect between MPS and Captex 300 in enhancing FEN aqueous solubilization was attained. It was concluded that a balance between in vitro performance and drug loading is key, and the optimum drug load was determined to be between 7–16% *w/w*, which corresponds to (200–400% equilibrium solubility in lipid S_eq_). This study provides valuable insight into the impact of key characteristics of SLHs, in constructing optimized solid-state lipid-based formulations for the oral delivery of PWSDs.

## 1. Introduction

Due to the staggering number of new drug entities with poor physicochemical properties under development, formulation scientists are challenged to design innovative formulations that successfully overcome these molecules’ inherent slow dissolution and poor oral absorption [1]. Amongst novel drug delivery systems, lipid-based formulations (LBFs) present opportunities for successfully delivering poorly water-soluble drugs (PWSDs), owing to their ability to mimic the postprandial effect [2]. Upon ingestion, the lipid content within these formulations triggers the release of digestive pancreatic and gallbladder enzymes and a sequence of digestive processes that result in the dynamic formation of a range of colloidal systems (e.g., micelles) [3,4]. This naturally occurring phenomenon maintains the drug in the solubilized state and creates a concentration gradient that acts as a driving force for absorption, thus reducing the food effect for lipophilic drugs. Furthermore, such formulations deliver the drug to the gastrointestinal tract (GIT) in a pre-solubilized form, eliminating the requirement for dissolution as a rate limiting step for absorption [5]. As such, LBFs present promising benefits for poorly water-soluble, highly permeable, BCS (Biopharmaceutics Classification System) class II drugs [6,7].

Numerous studies have shown that, by manipulating the lipids and excipients used, enhancements in permeability, intestinal solubilization and absorption through the lymphatic system can be achieved, along with benefits in bypassing first-pass metabolism, inhibition of efflux transporters, and prevention of pre-systemic metabolism [4,8,9]. Although considerable research has been devoted to examining and refining formulation approaches to enhance the biopharmaceutical performance of LBFs, the number of successful products on the market are limited [5]. This is mainly due to their low stability, tendency to recrystallize and precipitate in vivo and costly manufacturing processes. In addition, several studies have reported poor correlation between in vitro and in vivo results [10,11]. 

Solidification represents an effective solution to overcome challenges associated with liquid LBFs, while optimizing the advantages related to lipids [6,12,13]. Solid-state LBFs are fabricated by adsorbing the liquid-state LBFs onto a solid carrier material. This can be achieved using various solid carriers (silica and silicate materials, polysaccharide, polymeric, and protein) and/or by adapting different techniques, including spray drying, lyophilization, rotary evaporation, melt extrusion, and melt granulation [14,15,16]. Silica-based adsorbents are commonly used as solid carriers for LBFs due to their biocompatibility, inert nature and highly porous structure [17,18], which provides increased surface area for adsorption of liquid lipids [19,20]. Furthermore, the high degree of versatility afforded by silica materials introduces the ability to manipulate the biopharmaceutical performance of silica lipid hybrids (SLHs) through changes in particle size, nanostructure and porosity [17,21,22]. For example, several studies have verified the ability to control both the rate and extent of lipid digestion, and the subsequent rate of drug release and absorption in SLHs through changes in nanostructure and surface chemistry [23,24,25,26]. Therefore, careful examination of the physiochemical properties of the silica carrier and composition should be considered during the fabrication of solid-state LBFs in order to seize their full therapeutic potential. 

For most LBFs, typical drug loads are ≤5% *w/w* [24,27]. The drug loads are further reduced when LBFs are solidified by adsorption onto a solid carrier [16,19], such as for SLH, which in turn limits their potential commercial application to low dose, highly potent drugs. Thus, despite their success in stabilizing precursor liquid LBFs and significantly enhancing dissolution and solubilization of model drugs, low drug loading levels limit the commercial translation of SLHs. In addition to understanding the structure-activity relationships between porous silica properties and drug solubilization, recent attention has been afforded to improving the drug loading within SLHs through a supersaturation approach, which has led to the creation of supersaturated SLHs (super-SLH) [28,29,30,31].

Super-SLH, defined as drug dissolved in lipid above its equilibrium solubility (S_eq_) at room temperature, were fabricated by dissolving quantities of drug in a lipid using heat, followed by solidification employing preformed mesoporous silica microparticles, which upon cooling, generated supersaturated powders [30]. The lipid-drug solution was imbibed into the nanopores of the silica and/or stabilized on the surface of the silica particles to maintain the drug in a molecular state [31]. The method has demonstrated high drug loading, increased stability of the supersaturated drug, enhanced dissolution, and improved oral bioavailability of the PWSDs ibuprofen (IBU) [29,30] and abiraterone acetate (AbA) [28,31]. However, there is a fine balance between increasing the drug loading/supersaturation level and achieving enhanced biopharmaceutical performance, as the drug can recrystallize in the solid state, resulting in reduced dissolution. For IBU super-SLH, the ideal supersaturation level was 227% S_eq_, which achieved a 2.2-fold enhancement in oral bioavailability in Sprague-Dawley rats when compared to the commercial product, Nurofen [30]. In contrast, for AbA super-SLH, despite the supersaturated formulations significantly enhancing in vitro solubilization, only the unsaturated SLH (at 90% S_eq_) achieved an oral bioavailability that exceeded the commercial product Zytiga (1.4-fold) in Sprague-Dawley rats [28]. Furthermore, when AbA super-SLH contained different lipids (Capmul PG8 or Capmul MCM), there were significant differences between their loading and in vitro and in vivo performances.

The published literature on super-SLH highlights that their performance is highly drug- and lipid-dependent, with only two drugs and two lipids being investigated thus far. Furthermore, only one type of silica has been applied to super-SLH. Therefore, there is significant opportunity for further investigation into the influence of these excipients to develop improved super-SLH formulations and exploit their full potential. On this basis, the focus of this study was to investigate the impact of (i) porous silica nanostructure, (ii) lipid type, and (iii) drug loading on the in vitro solubilization, dissolution, and solid-state stability of super-SLH [26]. To investigate silica nanostructure, preformed mesoporous silica microparticles were compared to a bottom-up approach to prepare porous silica microparticles that could be loaded with liquid lipid through spray drying fumed silica (FS). The lipids Capmul PG8 (monoester of caprylic acid) and Captex 300 (medium chain triglyceride) were investigated, with drug loads corresponding to 80, 200, 400, and 600% S_eq_. Fenofibrate (FEN), an antilipemic agent and BCS class II compound, was selected as the model drug to be applied to super-SLH for this study. It was chosen due to its poor water solubility (<3 µg/mL at 37 °C), its extensive use as a model drug, well documented application to LBFs, and as the first neutral compound to be applied to super-SLH [27,28,29,30,31]. Furthermore, this study marks the first application of FS as a solid carrier that can be loaded with supersaturated liquid lipid in super-SLH. The key findings of this study provide a valuable insight into the role of porous silica nanostructure, lipid type, and drug loading, for constructing optimized solid-state LBFs for the oral delivery of PWSDs.

## 2. Materials and Methods 

### 2.1. Materials 

Fenofibrate (FEN) (≥99%, powder) was purchased from Sigma-Aldrich (Castle Hill, Australia). Commercially available standard APO-fenofibrate tablets (Apotex, Macquarie Park, Australia) were purchased from local suppliers and crushed into a powder before use. The lipids, Capmul PG8, Captex 300 and Capmul MCM, were sourced from Abitec (Columbus, OH, USA). Fumed hydrophilic silica nanoparticles (Aerosil 300 Pharma), with a surface area of 300 ± 30 m^2^/g were donated by Evonik (Melbourne, Australia). Mesoporous silica microparticles (Parteck SLC 500), with a particle size of 9–11 µm and pore size of 6 nm, and sodium lauryl sulfate (SLS) (used to prepare dissolution medium) were donated and purchased from Merck (Melbourne, Australia), respectively. Fasted state simulated intestinal fluid/fed state simulated intestinal fluid/fasted state simulated gastric fluid (FaSSIF/FeSSIF/FaSSGF) biorelevant powder, to prepare biorelevant media for gastrointestinal lipolysis, was obtained from Biorelevant (Biorelevant.com, London, UK). Porcine pancreatin extract was supplied by MP Biomedicals (Seven Hills, Australia). 4-bromophenylboronic acid (4-BBA) and lipase from *Candida antarctica* were purchased from Sigma-Aldrich (Castle Hill, Australia). HPLC-grade methanol was purchased from local suppliers. High purity Milli Q water (Merck Millipore, Bayswater, Australia) was used throughout the study.

### 2.2. HPLC Assay for Fenofibrate Quantification

Fenofibrate was analyzed using a Shimadzu high-performance liquid chromatograph (HPLC) system (Kyoto, Japan) equipped with a Phenomenex Kinetex^®^ 2.6 µm PS C18 column (250 × 4.6 mm) maintained at 40 °C. The mobile phase contained 90% methanol and 10% water and was eluted at a flow rate of 0.7 mL/min. The effluents were analyzed by a UV detector at 288 nm, and the retention time for FEN was approximately 5.8 min. The concentration of FEN in each sample was determined using calibration curves generated over the range of 0.05–10 µg/mL with R^2^ values > 0.999.

### 2.3. Lipid Solubility Studies

The equilibrium solubility (S_eq_) of FEN in Capmul PG8, Captex 300, and Capmul MCM was determined in triplicate at 25, 40, and 60 °C. An excess of FEN was added to centrifuge tubes containing approximately 0.5 g lipid. The tubes were vortex mixed for 10–20 s to aid drug dissolution and placed on a rotator in an oven at the required temperature. After 24 h, samples were centrifuged at 44,800 rcf for 30 min at the required temperature to precipitate any undissolved drug. Methanol was used to extract the dissolved drug from the supernatant and was appropriately diluted with mobile phase prior to HPLC analysis. Samples were vortexed and returned to the rotator in the oven and analyzed every 24 h until equilibrium solubility was reached. This was indicated by <10% change in consecutive solubility measurements.

Due to a temperature limit on the centrifuge, centrifugation was not possible at 60 °C. Therefore, samples were left in the oven to allow for undissolved drug to settle as a pellet, and aliquots were carefully taken from supernatant. This method was previously validated [29], and an acceptable standard deviation was attained.

### 2.4. Fabrication of FEN Loaded Super-SLH

#### 2.4.1. Preparation of Spray Dried Microparticles

In order to obtain porous silica microparticles, a suspension of fumed silica nanoparticles (5% *w/w*) was sonicated overnight to allow effective hydration and separation of the silica nanoparticles prior to spray drying with a Büchi Mini Spray Dryer B290 apparatus (Büchi, Flawil, Switzerland). Spray drying was performed under the following conditions: inlet temperature 160 °C, outlet temperature 90 °C, aspirator setting 100, pump set at 20%, and product flow rate 6 mL/min. Fumed silica nanoparticles were successfully spray dried to form porous silica microparticles (80% yield).

#### 2.4.2. Preparation of Super-SLH

This method was adapted from the previously established methods [29,30]. FEN was weighed into glass vials with 0.5 g of lipid to achieve concentrations equivalent to 80, 200, 400, and 600% S_eq_ at room temperature. The glass vials were placed in the oven at 60 °C for approximately 10–30 min with intermittent shaking to dissolve the drug. Half a gram of silica microparticles (either FS or MPS) was added into the glass vial and immediately physically mixed to allow the mixture to form a white free flowing powder and allowed to cool. A ratio of 1:1 *w/w* silica: lipid was maintained throughout the study. Formulations were freshly prepared and were tested within 24 h.

### 2.5. Physicochemical Characterization

#### 2.5.1. Solid-State Characterization

Crystallinity of encapsulated FEN was investigated using X-ray powder diffraction (XRPD) and differential scanning calorimetry (DSC) (TA instrument, Rydalmere, Australia). XRPD data was obtained using a Malvern Panalytical Empyrean XRPD (Malvern, UK). Samples were scanned between 5° and 90° (2θ) at a rate of 5 s/point and step size of 0.02°. For DSC analysis, samples were accurately weighed into aluminum pans and hermetically sealed with an aluminum lids and heated at a rate of 10 °C/min over a temperature range of 25–120 °C. XRPD and DSC measurements were taken 1 day after formulations were fabricated.

#### 2.5.2. Scanning Electron Microscopy (SEM)

The surface morphology of SLH was examined using scanning electron microscopy (SEM) (Zeiss Merlin, Oberkochen, Germany). Samples were applied to double-faced adhesive carbon tape and sputter coated with 10 nm platinum prior to imaging at an accelerating voltage of 1 kV.

#### 2.5.3. Confocal Laser Scanning Microscopy

A Zeiss Elyra PS-1 microscope (Jena, Germany) was used to perform confocal laser scanning microscopy on blank SLH. Silica microparticles were labeled with rhodamine B and lipids were labeled with coumarin 6. Confocal images were captured at the emission wavelength of 488 nm for coumarin 6 (excitation wavelength 497 nm) and 625 nm for rhodamine B (excitation wavelength 540 nm).

#### 2.5.4. Drug Load Determination

FEN content was determined by solvent extraction. Approximately 10 mg of formulation was weighed into a glass vial, followed by the addition of 10 mL of methanol. Vials were sonicated for 45 min to extract FEN. Aliquots were centrifuged (44,800 rcf for 20 min) to sediment undissolved solids. Then, the supernatants were appropriately diluted and analyzed using HPLC.

### 2.6. Aqeous Media Solubility Studies

The equilibrium solubility of FEN in 0.0125 M SLS, FaSSGF, and FaSSIF was determined by the addition of excess FEN to 1 mL of media in 1.5 mL centrifuge tubes. The tubes were vortexed for 10–20 s to aid drug dissolution and placed on a rotator in the oven at 37 °C. After 48 h, samples were centrifuged at 44,800 rcf for 30 min at 37 °C to precipitate any undissolved drug. Methanol was used to extract dissolved drug from the supernatant, prior to dilution with mobile phase and quantification of FEN content by HPLC.

### 2.7. In Vitro Dissolution Studies

FEN dissolution studies were performed to compare the dissolution kinetics of various SLH formulations, crystalline FEN and APO-fenofibrate. The studies were performed in 0.0125 M SLS solution, as FEN is a neutral drug and practically insoluble in aqueous media [32]. However, using SLS significantly enhances its solubility, and its use as a dissolution media has been evaluated for multiple FEN-containing formulations in order to achieve sink conditions [33,34,35,36]. Dissolution was performed using a Vankel USP II apparatus (Agilent Technologies, Santa Clara, CA, USA), 450 mL of media was maintained at 37 °C and rotating at 50 rpm. An equivalent of 10 mg of FEN of formulation was added to the dissolution vessel. Three-milliliter aliquots were removed at 1, 5, 10, 15, 30, 45, 60, and 90 min, replaced by fresh media, and filtered using 0.45 µm syringe filter prior to appropriate dilution with mobile phase and analysis with HPLC.

### 2.8. In Vitro Gastrointestinal Lipolysis Studies

For the present study, a combined gastrointestinal (GI) in vitro lipolysis model adapted from [37] was employed to test solubilization of FEN under digesting conditions. FaSSIF/FeSSIF/FaSSGF powder was used to prepare gastric (FaSSGF) and intestinal (FaSSIF) media, according to manufacturer’s protocol (biorelevant.com, London, UK). Media were freshly prepared before each experiment and used within 48 h, according to manufacturer’s recommendations.

#### 2.8.1. Preparation of Simulated Gastric Lipolysis Media

FaSSGF buffer (pH 1.6 ± 0.01) was prepared by dissolving 2.00 g NaCl in 1 L Milli Q water and adjusting the pH with 0.2 M HCl, prior to the addition of 0.06 g biorelevant powder to prepare biorelevant FaSSGF.

#### 2.8.2. Preparation of Simulated Intestinal Lipolysis Media

FaSSIF was prepared by dissolving 2.240 g FaSSIF/FeSSIF/FaSSGF powder in 1L pH 6.5 ± 0.01 buffer (0.42 g NaOH, 3.4 g NaH_2_PO_4_, 6.2 g NaCl, 1L MilliQ). NaOH (1 M) and HCl (1 M) were used to adjust the pH of the intestinal buffer. Following preparation and before use, intestinal lipolysis media was left standing for 2 h to allow equilibrium of buffer excipients and micelles formation. On the day of the experimental procedure, pancreatin extracts were prepared by stirring 2 g of pancreatin powder in 10 mL of intestinal lipolysis buffer (pH 6.5) for 15 min, followed by centrifugation (2268 rcf, 20 min, 4 °C). The supernatant was collected in a glass vial and kept refrigerated.

#### 2.8.3. Gastrointestinal Digestion Experimental Procedure

Lipid digestion kinetics were monitored for a total of 90 min (30 min gastric step followed by 60 min intestinal step). A Metrohm 902 Titrando pH-stat titration apparatus equipped with a Dosino 800 dosing apparatus (Herisau, Switzerland) was used for the studies. The experimental procedure was initiated with the addition of an amount of formulation corresponding to 3 mg of FEN into a temperature-controlled reaction vessel containing 10 mL of freshly prepared FaSSGF (37 °C, pH 1.6). Gastric lipolysis was carried out for 30 min and was initiated by the addition of 100 µL *Candida* lipase (600 tributyrin units lipase activity). Following gastric lipolysis, 18 mL of FaSSIF media was added (pH 6.5) to the reaction vessel, and the pH was automatically adjusted by the pH-stat apparatus to maintain pH at 6.5 ± 0.01. Following a pH-equilibration time of 3–5 min, intestinal lipolysis was initiated by the addition of 2 mL freshly prepared pancreatin extract, bringing the total volume to 30 mL. Fatty acids (FA) produced as a result of digestion were instantly titrated with 0.6 M NaOH to maintain a constant pH of 6.50 ± 0.01. The cumulative volume of NaOH was converted to the number of moles of NaOH consumed, which in turn corresponded to the number of moles of FA released. Data obtained from NaOH titration were used to compare the rate and extent of lipid digestion for the different formulations. It is important to note that NaOH titration during the gastric step does not reflect extent of lipolysis since the FA produced are protonated at pH 1.6 and could therefore not be recorded during gastric lipolysis [37].

#### 2.8.4. Drug Phase Partitioning During In Vitro Lipolysis

The solubilization of FEN in the aqueous phase during in vitro gastrointestinal lipolysis was measured. Aliquots (1 mL) were withdrawn at 1, 15, and 30 min (gastric phase) and 1, 5, 10, 15, 30, 45, and 60 min (intestinal phase) and transferred into 1.5 mL centrifuge tubes prefilled with 10 μL of 4-BBA (0.5 M in methanol) as a lipase inhibitor. Samples were centrifuged (32,760 rcf, 10 min, 25 °C) to separate the aqueous phase, containing solubilized FEN, and precipitated pellet. The supernatant was appropriately diluted prior to analysis by HPLC. The supernatant was discarded to leave the pellet and was re-dispersed through addition of 1 mL methanol, vortex mixed for 30 s, followed by sonication for 45 min. The tube was centrifuged (32,760 rcf, 10 min, 25 °C), and the supernatant was diluted and analyzed by HPLC.

### 2.9. Statistical Analysis

All statistical analyses of experimental data were performed using GraphPad Prism Version 8.0. Statistically significant differences were determined by one-way ANOVA followed by Tukey’s post-test for multiple comparisons. Values are reported as the mean ± standard deviation (SD), and the data were considered statistically significant when *p* < 0.05.

## 3. Results

### 3.1. Influence of Temperature on FEN Solubility in Lipids

**Figure 1:**
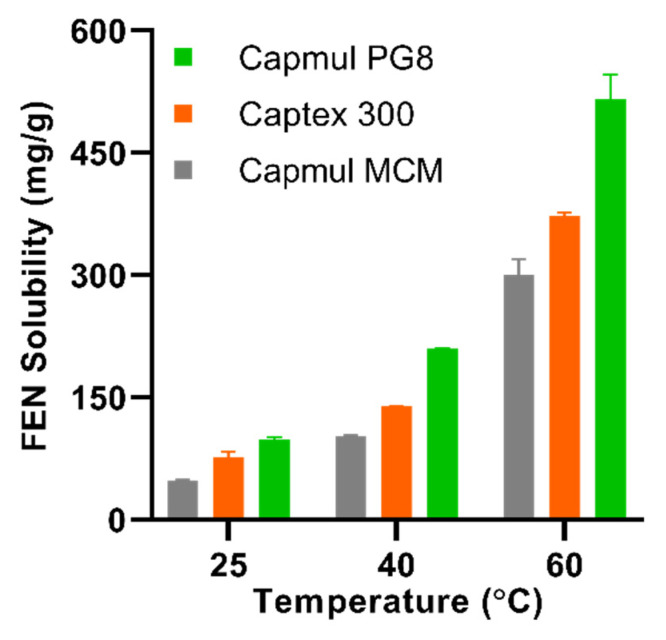
The temperature-dependent solubility of fenofibrate (FEN) in Capmul MCM (grey bars), Captex 300 (orange bars), and Capmul PG8 (green bars). Values represent mean ± SD, *n* = 3.

In order to select the optimum lipids for supersaturating FEN, solubility studies were performed at 25, 40, and 60 °C in Capmul PG8 (PG8), Captex 300 (C300) and Capmul MCM (MCM). It was evident that FEN solubility in all lipids increased considerably with temperature, achieving 4.4- to 7.7-fold greater FEN solubility at 60 °C compared to 25 °C (Figure 1). Regardless of temperature, FEN displayed greatest solubility in PG8, achieving a solubility of 98 ± 3.5 mg/g at 25 °C and 516 ± 29.5 mg/g at 60 °C. PG8 and C300 were chosen for the fabrication of FEN-loaded SLH due to both lipids exerting the highest drug loading at 60 °C. MCM was excluded due to its inferior drug loading capacity.

### 3.2. Super-SLH Preparation and Characterization

FEN-loaded SLH were fabricated with varying (i) lipid types, (ii) silica types, and (iii) drug loads. The compositions and drug loads of the fabricated formulations are summarized in Table 1. Subsequently, fabricated SLH formulations were identified and categorized according to type of lipid used to dissolve FEN (PG8 or C300), type of silica microparticles used to stabilize precursor liquid lipid (FS or MPS), and drug load based on % S_eq_ ranging from 80% S_eq_ (unsaturated) to 600% S_eq_ (highly supersaturated). Furthermore, liquid lipid formulations were prepared at 80% S_eq_ in both lipids, in order to compare the influence of solidification on drug release and solubilization. Drug loads ranging from 3.8 to 22.7% *w/w* were attained for PG8-containing formulations and 3 to 18.8 % *w/w* for C300-containing formulations. Furthermore, three formulations were randomly selected to evaluate the homogeneity of FEN throughout the formulation and to assess the drug loading efficiency (Table S1). All tested formulations displayed high drug loading efficiency of at least 94 ± 2.8% *w/w* with small standard deviation.

#### 3.2.1. Drug Crystallinity

**Figure 2:**
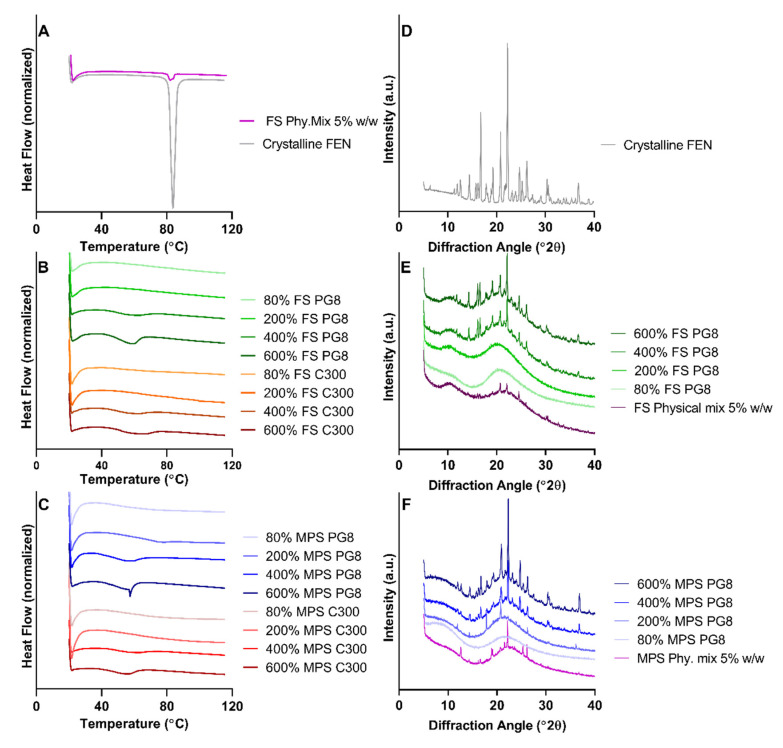
Differential scanning calorimetry (DSC) thermograms of (**A**) crystalline FEN and fumed silica (FS) physical mix 5% *w/w*, (**B**) FEN-loaded FS formulations and (**C**) FEN-loaded mesoporous silica (MPS) formulations. The X-ray powder diffraction (XRPD) diffractograms of (**D**) crystalline FEN, (**E**) FS PG8 formulations and (**F**) MPS PG8 formulations.

DSC and XRPD were utilized for the qualitative, solid-state characterization of FEN in the SLH formulations. The DSC thermograms of a physical mixture (5% *w/w* crystalline FEN with FS), crystalline FEN, FS, and MPS formulations are displayed in Figure 2. Crystalline FEN exhibited a sharp endothermic peak at 80 °C, corresponding to its melting point [38], whereas the FS physical mixture exhibited a small endothermic peak (Figure 2A). Furthermore, this thermogram suggests that crystalline FEN could be detected at concentrations ~5% *w/w* when present within silica particles. Regardless of the silica type or lipid used, endothermic peaks were absent for all 80% and 200% SLH formulations, suggesting that the drug was in a non-crystalline state. However, all other formulations of higher drug loads demonstrated shifted endothermic peaks varying in size and stretching between 45–60 °C. The shifted endothermic peaks for all 400% formulations were smaller than for the 600% formulations, which suggests the presence of higher concentrations of crystalline FEN at higher saturation levels (Figure 2B,C). No apparent difference in DSC thermograms was observed between PG8 or C300 SLH formulations, whether loaded in FS or MPS. Therefore, XRPD patterns were only obtained for SLH formulations containing PG8, since higher drug loads were achieved in comparison to the SLH C300 formulations.

Crystalline FEN demonstrated characteristic XRPD patterns as previously reported (Figure 2D) [39]. For the 5% *w/w* physical mixture, characteristic crystalline FEN patterns can be observed but differ in intensity between FS and MPS (Figure 2E,F). XRP diffractograms indicate that the method can detect as low as 5% *w/w* crystalline FEN. In SLH formulations fabricated with FS PG8, no crystalline FEN was detected at drug loads of 80% and 200%. However, at 400% and 600% FS PG8, intense peaks were observed, suggesting the presence of crystalline FEN. These findings correspond with data obtained with DSC analysis. However, all supersaturated MPS PG8 formulations (200, 400, and 600% S_eq_) displayed characteristic peaks of varying intensities, suggesting the presence of crystalline FEN (Figure 2F), and no crystalline drug was detected with unsaturated 80% P PG8 formulation. It is important to note that some characteristic peaks where absent from XRPD pattern obtained from 200% P PG8, suggesting the presence of a FEN polymorph [38]. For both FS and MPS systems, XRPD patterns obtained from 600% S_eq_ formulations displayed peaks with higher intensities than corresponding 400% S_eq_ formulations, displaying an ~2-fold increased intensity, suggesting the presence of higher amounts of crystalline FEN in SLH prepared at 600% S_eq_. Furthermore, MPS formulations exhibited peaks with higher relative intensities compared to corresponding FS formulations that were ~1.6-fold higher at 400% and 600% S_eq_. This was evident when the peak intensities obtained at 22.3° (2θ) were plotted against % S_eq_, as displayed in Figure S1.

#### 3.2.2. Surface Morphology and Lipid Distribution

**Figure 3:**
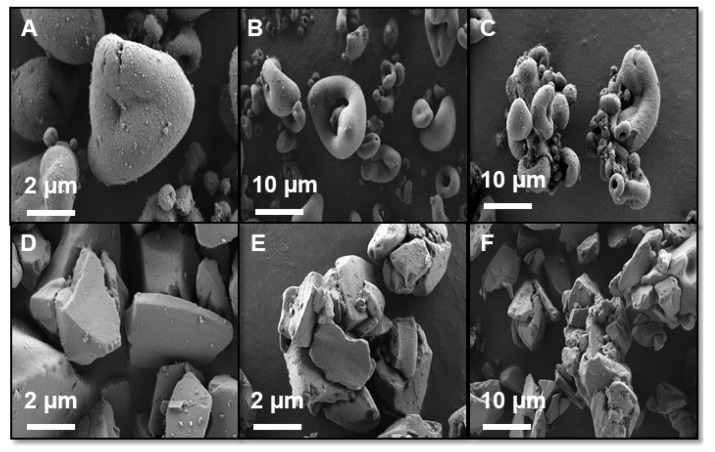
SEM images of (**A**) blank FS, (**B**) 80% FS PG8, (**C**) 200% FS PG8, (**D**) blank MPS, (**E**) 80% MPS PG8, and (**F**) 200% MPS PG8.

**Figure 4:**
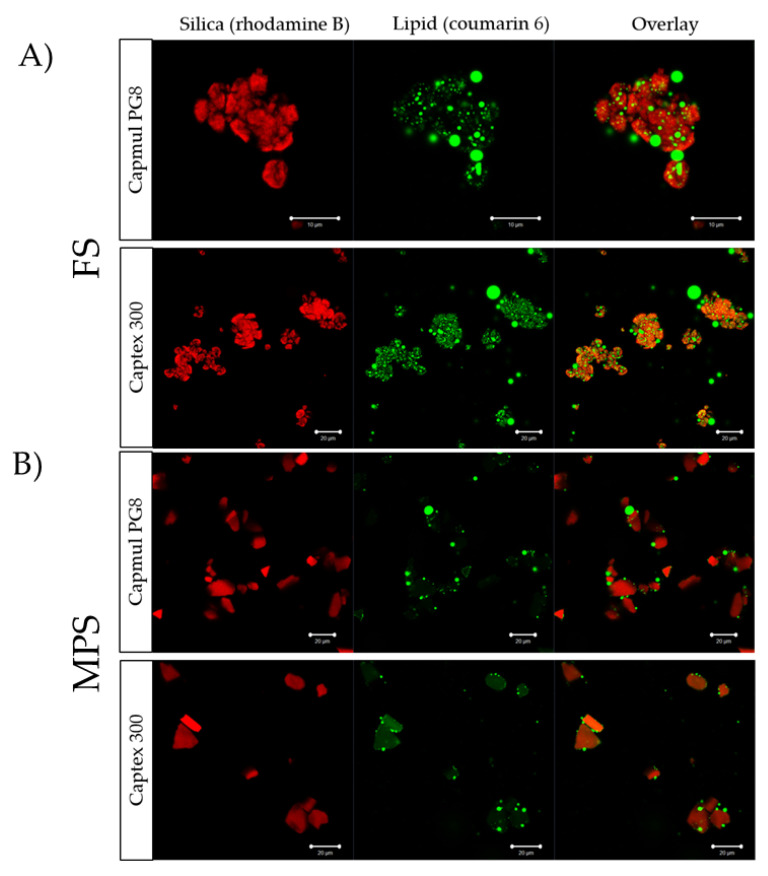
Confocal images of: (**A**) SLH prepared with FS and (**B**) SLH prepared with MPS. Rhodamine B (red) was used to label silica, and coumarin 6 (green) was used to label lipid.

Surface morphology, internal structure, and lipid distribution within fabricated SLH formulations were examined using SEM and confocal laser scanning micriscopy (Figure 3 and Figure 4). Blank FS particles formed semi-spherical shapes of varying particle sizes with distinct concave-like structures (Figure 3A). No difference in appearance was observed between blank FS and 80% FS PG8 (Figure 3B). However, at 200% saturation level, regardless of the lipid used, FS particles appeared to form clusters and large aggregates (Figure 3C). In contrast, MPS appeared as angular structures and formed aggregates irrespective of the lipid used or FEN saturation level (Figure 3E,F). No difference in appearance was observed between SLH prepared with either lipid types; therefore, only SEM images of SLH prepared with PG8 are shown.

Confocal imaging revealed that the lipid distribution within the silica microparticles was dependent on the type of silica and lipid used. More lipid was observed within the internal structure of FS particles than MPS, evidenced by the intensity of the fluorescently labeled lipid within the pores of the silica microparticles (Figure 4). However, it was evident that PG8 was less prone to imbibe into the porous matrix compared to C300, with lipid droplets tending to reside between inter-particle cavities, irrespective of the silica type. In contrast, C300 homogeneously distributed throughout the internal structure, with the presence of additional lipid droplets between the particles.

### 3.3. In Vitro Dissolution

**Figure 5:**
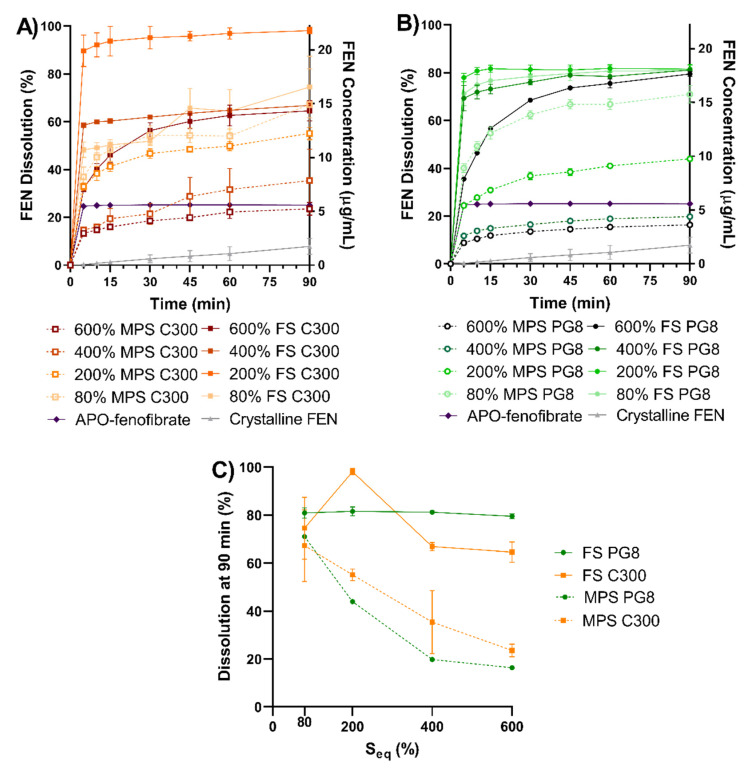
The in vitro dissolution profiles of crystalline FEN, APO-fenofibrate and FEN-loaded SLH formulations (at different saturation levels) in 0.0125 M SLS, dosed at 10 mg of FEN over 90 min at sink conditions. (mean ± SD, *n* = 3). (**A**) SLH C300 formulations, (**B**) SLH PG8 formulations, and (**C**) FEN % dissolution at 90 min vs % S_eq_.

The dissolution performance of various SLH prepared with FS and MPS, APO-fenofibrate, and crystalline FEN is reported in Figure 5. The equilibrium solubility of FEN in 0.0125 M SLS (0.36%) was 128 ± 0.01 µg/mL; thus, the study was performed under sink conditions. Crystalline FEN and APO-fenofibrate displayed 7.8% and 25.1% solubilization after 90 min, respectively. At 80% saturation level, all SLH formulations, irrespective of type of silica or lipid, exhibited significantly enhanced FEN dissolution, with FS formulations achieving 70.9–98.1% after 90 min and displaying slightly improved performance compared to MPS formulations (*p* > 0.05). FEN dissolution from FS formulations at 80% S_eq_ corresponded to 8- to 10-fold and 2.6- to 3.2-fold increase in dissolution compared to crystalline FEN and APO-fenofibrate, respectively (*p* < 0.05). However, FEN dissolution from supersaturated SLH formulations was dependent on type of lipid and silica utilized. FS PG8 formulations at all saturation levels (80%, 200%, 400%, 600% S_eq_) achieved a comparable extent of FEN dissolution of ~80% after 90 min, despite 600% FS PG8 displaying slower release kinetics (*p* > 0.05) (Figure 5B). In contrast, FEN dissolution was significantly hindered in supersaturated MPS PG8 formulations, with 200% MPS PG8 achieving 43.9% FEN dissolution after 90 min, and was further reduced for 400% and 600% S_eq_ with no significant difference compared to crystalline FEN and APO-fenofibrate (*p* > 0.05).

The 200% FS C300 formulation achieved the highest FEN dissolution of 98.1%, which corresponds to approximately 4- and 14-fold increased dissolution compared to APO-fenofibrate and crystalline FEN, respectively (*p* < 0.05). However, this was not statistically significant when compared to 200% FS PG8 (*p* > 0.05). Only 66.8% and 64.6% FEN dissolution was observed from 400% and 600% S_eq_ for FS C300 formulations, respectively. When MPS was utilized to solidify supersaturated C300 LBFs, a similar pattern was observed to supersaturated MPS PG8 formulations (Figure 5C), where the dissolution was significantly reduced with increased supersaturation. 200% MPS C300 achieved 55.2% FEN dissolution, whereas FEN dissolution of only 35.4% and 23.5% was attained from 400% and 600% MPS C300 formulations, respectively, with no significant difference compared to crystalline FEN and APO-fenofibrate (*p* > 0.05). The influence of silica type and saturation level on FEN dissolution was evident when FEN dissolution at 90 min was plotted against saturation level (S_eq_). As displayed in Figure 5C, SLH prepared with FS, irrespective of type of lipid or % S_eq_, achieved similar extent of dissolution (*p* > 0.05). In contrast, MPS stabilized SLH formulations displayed significant linear reduction in FEN dissolution with an increase in % S_eq_. The decrease in performance was more profound for MPS PG8 formulations (R^2^ = 0.86) than MPS C300 formulations (R^2^ = 0.80); however, it was not statistically significant (*p* > 0.05).

### 3.4. In Vitro Lipolysis and Aqueous Solubilization

#### 3.4.1. Aqueous Solubilization

The partitioning of FEN between the aqueous phase and the pellet was measured over 90 min during two-phase gastrointestinal lipolysis. The total percentage of FEN recovered ranged between 70 to 98% for all formulations (Figures S2 and S3). Difficulty in isolating the pellet and possible pellet loss during the separation of the aqueous phase and pellet have contributed to having less than 100% total recovered FEN. This issue has been previously reported and was attributed to either drug loss during the separation process or inability to analyze drug retained in the undigested oil phase [28].

**Figure 6:**
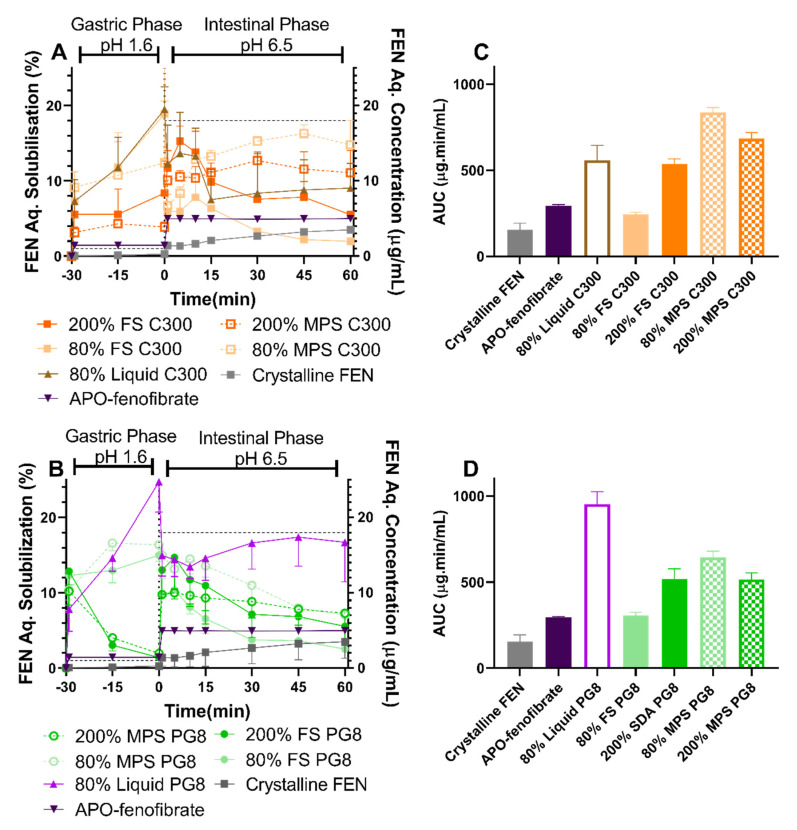
FEN aqueous concentration-time profiles from crystalline FEN, APO-fenofibrate, and FEN-loaded SLH formulations, in fasted state simulated gastric fluid (FaSSGF) (30 min) and fed state simulated intestinal fluid (FaSSIF) (60 min) under digesting conditions, dosed at 3 mg of FEN (mean ± SD, *n* = 3). (**A**) SLH C300 formulations and (**B**) SLH PG8 formulations. Dotted line represents the FEN S_eq_ in biorelevant media. The area under the curve (AUC) of FEN aqueous solubilization-time profile measured during the intestinal phase from (**C**) crystalline FEN, APO-fenofibrate, and SLH C300 formulations and (**D**) crystalline FEN, APO-fenofibrate, and SLH PG8 formulations.

The aqueous concentration-time profiles of solubilized FEN during in vitro gastrointestinal lipolysis are displayed in Figure 6A,B. Crystalline FEN displayed negligible solubilization during the gastric phase, while achieving only 3.5% solubilization after complete intestinal lipolysis. For APO-fenofibrate, once re-dispersed in simulated intestinal digesting media, 5% of total FEN was immediately solubilized with no further changes in solubilization over the entirety of the lipolysis period. The characteristic precipitation of FEN from LBFs caused difficulties in data interpretation and distinguishing enabling formulations. However, the extent and occurrence of FEN precipitation was influenced by the silica nanostructure, type of lipid and drug load evident in patterns observed from the aqueous solubilization-time profiles and area under the intestinal solubilization-time curve (AUC) reported in Figure 6. It is important to note that SLH formulations prepared at 400% and 600% S_eq_ did not improve FEN dissolution (Figure 5); therefore, these formulations were not pursued further in aqueous solubilization studies.

When comparing solidified formulations to 80% liquid lipid, enhanced performance was observed with 80% liquid PG8 during the gastric step, achieving the highest FEN solubilization of 25%, after 30 min (*p* < 0.05). Despite FEN precipitation during the intestinal phase between 1–5 min, FEN solubilization gradually increased to reach 18%, after 90 min, which was significantly higher than all SLH PG8 formulations (*p* < 0.05). However, 80% liquid C300 formulation displayed an enhanced FEN aqueous solubilization profile during the intestinal phase compared to corresponding solidified 80% FS C300 but lower performance than corresponding 80% MPS C300 (*p* < 0.05) (Figure 6A,C).

For FEN solubilization from SLH formulations prepared at 80% S_eq_, FEN precipitation was triggered by the transition into the intestinal phase at time points ranging between 1–5 min and was followed by a period of slightly enhanced solubilization prior to another decrease in solubilization after 15 min. During the gastric step, 80% FS PG8, 80% MPS PG8, 80% FS C300, and 80% MPS C300 formulations displayed enhanced FEN solubilization of 15%, 16%, 19%, and 12.4%, after 30 min, respectively (*p* > 0.05). This corresponds to 3.5- to 5-fold and 3- to 3.7-fold significantly enhanced solubilization compared to crystalline FEN and APO-fenofibrate, respectively (*p* < 0.05) (Figure 6A,B). Transition into the intestinal phase triggered FEN precipitation where the percentage FEN solubilized decreased to 13% and 6.7% from 80% MPS PG8 and 80% MPS C300, respectively. However, a more dramatic drop in FEN solubilization to 9% and 5.9% was observed from 80% FS PG8 and 80% FS C300 formulations, respectively, at 1 min of the intestinal phase. Furthermore, 80% MPS C300 was able to gradually enhance FEN solubilization and prevent its precipitation, achieving 16% FEN solubilization, after 90 min which was significantly greater than FEN solubilization achieved by corresponding 80% FS C300 formulation (*p* < 0.05).

In contrast, FEN solubilization behavior from super-SLH (200% S_eq_) was dependent on the type of lipid utilized, where a decrease in FEN solubilization was attained in the gastric phase for 200% FS PG8 and 200% MPS PG8 followed by enhanced solubilization in the intestinal phase achieving 15% and 10% solubilization, respectively (Figure 6C). FEN solubilization was maintained at 4–5% during the gastric phase by 200% FS C300 and 200% MPS C300 formulations followed by enhanced FEN solubilization in the intestinal phase, achieving 15.3% and 10.4%, after 5 min, respectively, prior to FEN precipitation by only 200% FS C300, at 15 min, dropping FEN solubilization to less than 10%. In contrast, FEN solubilization from 200% MPS C300 gradually increased during the intestinal phase achieving 12.7%, after 90 min. This corresponds to 3.6- and 2-fold enhanced solubilization compared to crystalline FEN and APO-fenofibrate, respectively.

All SLH formulations achieved FEN solubilization above FEN equilibrium solubility in FaSSGF (1.1 ± 0.4 µg/mL) during the gastric phase and below its equilibrium solubility in FaSSIF during the intestinal phase (18.1 ± 0.8 µg/mL). Regardless of lipid type, a pattern of enhanced FEN solubilization with increased drug load in FS formulations was clearly evident in data obtained from AUC of FEN solubilization-time profile during the intestinal phase (Figure 6C,D), whereas a decrease in performance with supersaturation was observed for MPS formulations. It was evident that 80% MPS C300 improved the aqueous solubilization of FEN when compared to the 80% liquid C300. In contrast, for PG8 formulations, the presence of both silica types reduced FEN aqueous solubilization when compared to 80% liquid PG8.

#### 3.4.2. Lipid Digestion

**Figure 7:**
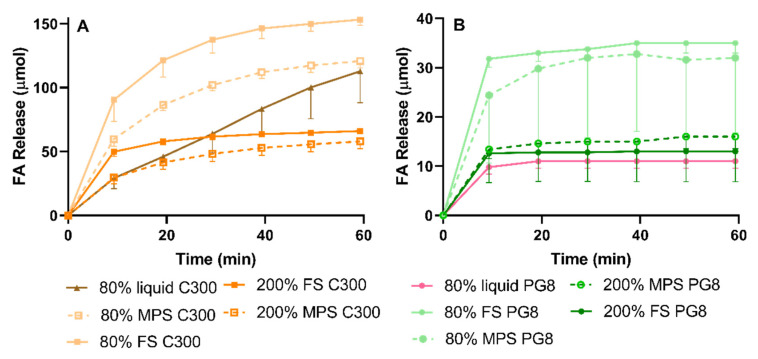
Fatty acid (FA) release-time profile from: (**A**) C300 formulations and (**B**) PG8 formulations, during the intestinal phase of in vitro gastrointestinal (GI) lipolysis in FaSSIF for 60 min, dosed at 3 mg of FEN (mean − SD, *n* = 3).

The lipid digestion profiles during the intestinal phase of the gastrointestinal lipolysis of all investigated LBF are displayed in Figure 7. Crystalline FEN and APO-fenofibrate did not display any lipid digestion as they did not contain lipid (data not shown). The fabricated formulations differed in their lipid content per dose, as dosing was based on the amount of formulation equivalent to 3 mg of FEN. The amount of lipid dosed ranged from 19.5–48.7 mg (Table S2). The 80% liquid PG8 formulation exhibited the lowest extent of digestion compared to its corresponding solidified SLH formulation (FS or MPS). In contrast, 80% liquid C300 displayed a slow gradual increase in lipolysis over 60 min, but the final extent of lipolysis was lower than corresponding SLH formulations. The amount of digestion decreased as a function of increasing drug load, since all 80% SLH formulations displayed significantly higher extent of digestion when compared to their corresponding 200% formulations, regardless of the type of lipid (*p* < 0.05). FA released from C300 SLH formulations were 5-fold greater than corresponding SLH PG8 formulations, regardless of the type of silica or drug load. At the same saturation level and lipid type, FS facilitated greater extent of lipolysis when compared to corresponding MPS formulations; however, the difference was not statistically significant (*p* > 0.05).

## 4. Discussion

Numerous studies have shown that small changes in the nanostructure, surface chemistry and composition of SLH particles can significantly impact lipid digestion dynamics, which in turn influences drug solubilization and release kinetics [23,40]. Therefore, the aim here was to examine the influence of (i) silica nanostructure, (ii) type of lipid, and (iii) drug load on the in vitro solubilization and solid-state stability of the model drug FEN when incorporated into an SLH formulation.

It is well known that spray drying is amongst the techniques commonly employed to solidify liquid-state LBFs and is recognized to generate highly porous three-dimensional nanostructured matrices with superior physicochemical properties [14]. Here, spray drying was employed to generate porous silica microparticles (FS) from precursor fumed silica nanoparticles suspensions, prior to drug/lipid encapsulation via adapting the simple mixing technique [29]. Supersaturation was achieved by adding drug above its equilibrium saturation level in either of two lipids (PG8 or C300) using heat (≤60 °C) [29]. The generated, thermodynamically unstable, supersaturated liquid lipids were adsorbed into the nanopores or onto the surface of FS or MPS to allow for direct comparison.

### 4.1. Impact of Drug Loading

All synthesized formulations were found to be homogeneous and achieved high FEN loading efficiency regardless of the type of silica or lipid. Thus, this validates the efficiency of the simple mixing technique in minimizing drug/lipid loss during the fabrication process. The small amount of drug loss (<6% *w/w*) (Table S1) can be attributed to lipid solution of the drug sticking to the vial and not being adsorbed into the nanopores.

Both FS and MPS, silica microparticles were able to maintain FEN in a non-crystalline state at a sub-saturation level of 80% S_eq_, regardless of the lipid type. DSC and XRPD data confirmed the presence of a greater extent of FEN crystallinity in all 600% S_eq_ formulations, compared to 400% S_eq_ (Figure 2). This correlated with reduced in vitro dissolution performance (Figure 5). Furthermore, FEN dissolution and aqueous solubilization from unsaturated (80% S_eq_) and super-SLH (200, 400 and 600% S_eq_) was dependent on the type of silica and lipid utilized. The overall trend was that an increase in drug load resulted in an increase in FEN crystallization and aggregation of the silica microparticles.

It is important to note that, for FEN-SLH formulations, the endothermic peak observed in DSC thermographs were at a lower temperature than the melting point of crystalline FEN. The depression in melting point was previously reported in IBU-loaded super-SLH formulations and was attributed to the confinement of the drug crystallites in the nanopores [29]. The authors postulated that the presence of the drug outside of the pores and on the surface on the silica will not cause any depression in melting point, which implies the successful imbibition of drug and lipid into the nanopores of the silica microparticles. Based on this hypothesis and supported by XRPD data, it was confirmed that crystalline FEN is present in the formulations where display shifted endothermic peaks at 45–60 °C. Furthermore, it was hypothesized that the heating process during DSC analysis caused crystalline FEN present to re-dissolve in the lipid component of the SLH formulations displaying a shifted endothermic peak. However, further investigations are required to fully validate this hypothesis.

### 4.2. Impact of Silica Nanostructure

FS exhibited superior stabilization capacity and was able to stabilize a FEN saturation level of 200% S_eq_. It was evident that saturation levels greater than 400% S_eq_ surpassed silica’s stabilization capability and was reflected in DSC thermograms and XRP diffractograms. The relative intensities of XRPD patterns of MPS and FS formulations propose the presence of greater amounts of crystalline FEN in MPS compared to FS formulations, suggesting the nanostructured matrix of silica microparticles influences the physical state of the drug. XRPD is highly sensitive to molecular packing; therefore, any changes in the internal structure of the drug can be reflected in the patterns obtained [39]. This was evident in the XRPD pattern of 200% MPS PG8 formulation, suggesting the presence of FEN in a polymorphic form. However, the identification and confirmation of the presence and stability of FEN in any polymorphic form needs to be further investigated. Therefore, 200% S_eq_ was considered optimal in combination with FS as a solid carrier to maximize drug load while maintaining FEN in a solubilized non-crystalline state. These findings were in agreement with previous studies where super-SLH containing IBU and AbA displayed an increased amount of crystalline drug with increase in drug loading and optimum drug loading, which was 227% for IBU and 150% for AbA [28,31].

During in vitro dissolution studies (Figure 5), unsaturated (at 80% S_eq_) MPS and FS formulations had a comparable performance, regardless type of lipid used. In contrast, both the rate and extent of dissolution from super-SLH (200%, 400%, and 600% S_eq_) were significantly enhanced when using FS compared to corresponding MPS formulations. At higher saturation levels (400% and 600% Seq), it was evident that FS was able to maintain excellent FEN dissolution despite the presence of “crystalline” FEN and less amount of lipid in the formulations. However, the relatively larger amount of crystalline FEN present at 600% FS PG8 and 600% FS C300 resulted in slower release kinetics. In contrast, a reduction in dissolution performance was observed by MPS formulations with an increase in drug load. These findings were consistent with data obtained from FEN aqueous solubilization during in vitro lipolysis, despite the complexity of the data generated due to FEN precipitation.

FEN is a commonly used model PWSD in LBFs, and its tendency to precipitate from LBFs has been previously reported in multiple studies [11,41,42,43]. In these studies, authors proposed that, as digestion proceeds, formulations lose their solubilization capacity along with dilution of formulation triggered by the transition into intestinal digestion, where FEN precipitates with increasing FA concentration [41]. However, in these studies, FEN precipitation during in vitro lipolysis did not correlate with poor in vivo performance. Thomas et al. observed more extensive in vitro FEN precipitation from supersaturated self-nanoemulsifying drug delivery systems (super-SNNEDS) than from unsaturated SNEDDS [11]. Nevertheless, all SNEDDS formulations displayed enhanced in vivo bioavailability. FEN was shown to precipitate in a crystalline form in vitro but the extent of FEN precipitation in vitro was not known. These studies indicate that in vitro lipolysis models are not predictive of in vitro performance of FEN in a LBFs and should be used carefully to interpret such data [42].

In a previous study, in order to understand the underlying mechanism of FEN absorption and possible precipitation in vivo, orlistat as a lipase inhibitor was co-administered with SNEDDS containing FEN [43]. The study showed that lipase inhibition did not influence FEN bioavailability from SNEDDS, therefore digestion does not impact FEN absorption but can be beneficial when FEN is present in crystalline form in the formulation. However, it was still not known whether FEN precipitation occurs in vivo and to what extent. Recently, Tanaka et al. [44] found that FEN precipitated in the stomach of rats in an amorphous form with 20% microemulsion (ME) and crystalline form with 90% ME. In both cases, rapid redissolution was attained in the duodenum and 90% ME achieved significantly enhanced bioavailability. It is important to note that precipitation was more pronounced in the closed in vitro lipolysis system, which implies the importance of adding an absorption mimicking step [45]. The authors concluded that precipitation from the supersaturated state in the gastrointestinal environment was suppressed by the rapid absorption process for a highly permeable drug, like FEN, creating an absorption sink that acts as a driving force for absorption. In addition, the authors suggested the possibility of enhanced solubilization capacity of GI fluid caused by the formation of colloidal species upon digestion of the lipid. Therefore, formulations that cause higher supersaturation in the GI fluid [46] and the inclusion of medium chain triglycerides might result in enhanced bioavailability, despite their tendency to precipitate in vitro. However, such conclusions cannot be made for this study as further in vitro studies are required to validate the full potential of the fabricated SLH formulations.

In the present study, a higher extent of lipid digestion, as reflected in FA release, correlated with more pronounced FEN precipitation. This was evidenced by the 80% liquid PG8 formulation exerting the greatest FEN solubilization but reduced FA release. Liquid lipid formulations investigated in this study formed large emulsion droplets during in vitro lipolysis, providing less surface area for lipase action. However, when lipid was internalized within a porous silica matrix, a high surface area for lipase-mediated digestion was provided [40], which led to a more pronounced FA release and subsequent FEN precipitation. In the case of SLH prepared by FS, supersaturation resulted in enhanced FEN solubilization. In contrast, SLH prepared with MPS achieved the best performance when unsaturated. This was in agreement with solubilization data and previous studies where AbA-loaded super-SLH (prepared with MPS) compromised its solubilization performance during lipolysis when compared to unsaturated SLH [25]. However, this was not the case for IBU (dissolution studies), which implies that the benefits of super-SLH are drug dependent and require further investigation and optimization.

**Figure 8:**
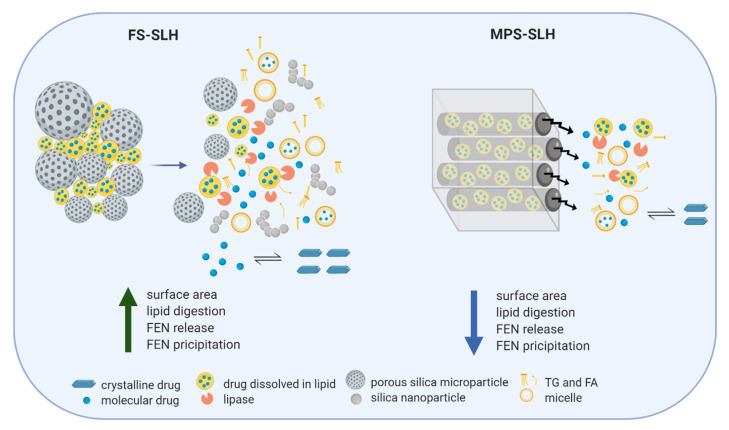
A schematic representation of the proposed mechanism of FEN release and precipitation from SLH made with FS and MPS, during in vitro lipolysis. Created with BioRender.com.

The difference in performance achieved by FS and MPS formulations can be attributed to their unique structure and resulting dynamic microenvironment when re-dispersed in biorelevant media (Figure 8). It is hypothesized that SLH microparticles prepared with FS de-aggregate and dissociate to their precursor silica nanoparticles upon dispersion in aqueous media, providing high surface area for lipase mediated digestion, dynamic mixed micelle formation, and subsequent drug release. For FEN, this results in a more profound precipitation in the closed in vitro digestion model, which lacks an absorption step. In contrast, FEN-loaded lipid droplets need to diffuse out of the 6 nm [29] structured nanopores of the MPS particles. This slows the kinetics of lipid/drug release, hindering lipolysis, which in turn leads to less pronounced FEN precipitation. In addition, MPS formulations were aggregated at all FEN saturation levels, suggesting poor lipid encapsulation which further reduced the surface area available for lipase action.

### 4.3. Impact of Lipid Type

FEN precipitation from SLH formulations occurred either during the gastric phase or during the intestinal phase depending on drug load and type of lipid used. Unlike dissolution, this made discrimination between formulations challenging. However, data generated display the benefits provided by super-SLH evident in the enhanced performance of all SLH formulations when compared to crystalline drug and APO-fenofibrate.

Lipids utilized in this study differ in their chemical composition and the physical interaction when imbibed into the silica nanopores. PG8 consists of propylene glycol (PG) mono (>90%)- and di (<10%)-esters of caprylic acid, while C300 is a medium-chain triglyceride manufactured by the esterification of glycerin and fatty acids, mainly caprylic (≈70%) and capric acid [47]. This impacts their physical encapsulation within the silica nanopores, lipolysis kinetics, digestibility, and the arrangement of digestion products [40]. Subsequently, influencing drug release and solubilization. Therefore, at the same lipid dose, C300 can generate greater FA release when digested compared to PG8. This was reflected in the FA release-time profiles. Conversely, the greater extent of digestion observed in C300 formulations did not correlate with enhanced FEN solubilization as no significant differences in solubilization capacity between SLH C300 and corresponding SLH PG8 formulations were observed.

Lipid distribution within the particles was different for each lipid. As evidenced in the confocal images (Figure 4), more PG8, compared to C300, was adsorbed on the surface of the silica microparticles than the pores with large droplets in between the particles, suggesting incomplete lipid loading that, in turn, can lead to aggregation, as observed in SEM images (Figure 3) and more evident with supersaturated FEN formulations. The distribution profile of PG8 within the SLH was similar to what was previously reported by Schultz et al. [31]. However, unlike findings in this study, the solubilization capacity of all SLH prepared with PG8 was much greater than those prepared with Capmul MCM, despite its even distribution within the silica matrix. In addition, it is important to highlight the synergistic effect obtained by the slow release kinetics of MPS along with higher amount of digestion products produced from the digestion of C300 in 80% and 200% MPS C300 formulations. As evidenced by the enhanced solubilization of FEN, these were the only SLH formulations to significantly enhance FEN solubilization between 15–60 min during the intestinal phase of in vitro GI lipolysis (Figure 6).

In the present work, we were able to highlight the importance of understanding the nanostructured matrix and composition of SLH microparticles in order to seize their full potential in enhancing the biopharmaceutical performance in delivering PWSDs, such as FEN. Each type of silica provides competitive advantages that can be tailored according to the outcome desired from the formulation. It has become evident that changes in the nanostructure and surface chemistry of SLH particles, even if small, can have significant impact on lipase activity which, in turn, influences drug release kinetics and absorption. Therefore, Future work will be directed toward understanding the influence of internal silica particles nanostructure, microporosity, and surface area on drug solubilization.

## 5. Conclusions

The role of key SLH characteristics on in vitro performance of FEN-loaded SLHs and super-SLH were investigated and compared. It was evident that the silica’s internal nanostructure influences lipase accessibility, which, in turn, affects the mechanism of drug release and FEN precipitation behavior. The crystalline FEN present at higher drug loads correlated with poor in vitro performance. Furthermore, the type of lipid utilized to dissolve the drug can impact its encapsulation within silica microparticles and the extent of digestion. When supersaturated, all FS formulations significantly enhanced FEN in vitro dissolution and aqueous solubilization compared to corresponding MPS formulations, crystalline FEN, and APO-fenofibrate. The implications of the main findings suggest that balance between high drug load and performance is key. Therefore, we can conclude that the optimum FEN loading is between 7–16% *w/w*, which corresponds to 200–400% S_eq_. GI lipolysis served as an important test to investigate FEN precipitation in vitro; therefore, future studies with absorption capabilities will be of great benefit to improve the understanding of this trend. Furthermore, the use of C300, a medium chain triglyceride, has proven to be beneficial in enhancing the aqueous solubilization of FEN and demonstrated synergistic action with MPS displaying best aqueous solubilization at 80% S_eq_, under digesting conditions. This was attributed to C300’s ability to generate a larger amount of digestion species along with the slow release kinetics from the restricted pores of MPS, resulting in enhanced FEN solubilization and less precipitation. However, further in vivo investigations are required to validate its full potential. The findings of this research demonstrate the impact of SLH structure and composition in fabricating optimized solid-state LBFs for the oral delivery of PWSDs; since the various silica and lipid types provide competitive advantages, formulations can be tailored depending on the release profile or outcome desired.

## Figures and Tables

**Table 1:** The calculated composition of the fabricated FEN-loaded liquid and silica-lipid hybrid (SLH) formulations.

Lipid Type	Silica Type	Formulation Name	Saturation Level (% S_eq_)	Drug Load (% *w/w*)	Lipid Load (% *w/w*)	Silica Load (% *w/w*)
Capmul PG8	-	80% Liquid PG8 *	80	7.3	92.7	-
	FS	80% FS PG8	80	3.8	48.1	48.1
		200% FS PG8	200	8.9	45.5	45.5
		400% FS PG8	400	16.4	41.8	41.8
		600% FS PG8	600	22.7	38.6	38.6
	MPS	80% MPS PG8	80	3.8	48.1	48.1
		200% MPS PG8	200	8.9	45.5	45.5
		400% MPS PG8	400	16.4	41.8	41.8
		600% MPS PG8	600	22.7	38.6	38.6
Captex 300	-	80% Liquid C300 *	80	5.8	94.2	-
	FS	80% FS C300	80	3	48.5	48.5
		200% FS C300	200	7.1	46.4	46.4
		400% FS C300	400	13.4	43.3	43.3
		600% FS C300	600	18.8	40.6	40.6
	MPS	80% MPS C300	80	3	48.5	48.5
		200% MPS C300	200	7.2	46.4	46.4
		400% MPS C300	400	13.4	43.3	43.3
		600% MPS C300	600	18.8	40.6	40.6

* Liquid formulations do not contain any silica (solid carrier).

## Supplementary Materials

The following are available online at https://www.mdpi.com/1999-4923/12/7/687/s1, Table S1: Drug loading efficiencies of 3 FEN-loaded formulations (values represent mean ± SD, *n* = 3), Table S2: The lipid content of the formulations and lipid dosed to lipolysis vessel. Figure S1. The relationship between XRPD peak intensity at 22.3° (2θ) and supersaturated drug loading (Seq) for FS PG8 formulations and MPS PG8 formulations, Figure S2. The % phase partitioning of FEN between the aqueous phase and pellet over 90 min after a 3 mg FEN dose of 80% formulations, Crystalline FEN and APO-fenofibrate, under biorelevant gastric and intestinal conditions. Values represent mean ± SD, *n* = 3 and Figure S3. The % phase partitioning of FEN between the aqueous phase and pellet over 90 min after a 3 mg FEN dose of supersaturated formulations, under biorelevant gastric and intestinal conditions. Values represent mean ± SD, *n* = 3.
