# Supplementary material for: Porous Nanostructure, Lipid Composition, and Degree of Drug Supersaturation Modulate In Vitro Fenofibrate Solubilization in Silica-Lipid Hybrids
Source: Pharmaceutics. 2020 Jul 21;12(7):687. doi: 10.3390/pharmaceutics12070687 (PMC7408050; doi:10.3390/pharmaceutics12070687)
Supplement: Supplementary file 1 [file pharmaceutics-12-00687-s001.zip › pharmaceutics-872038-supplementary.docx]

**Supplementary Materials**

Porous Nanostructure, Lipid Composition and Degree of Drug Supersaturation Modulate *In Vitro* Fenofibrate Solubilization in Silica-Lipid Hybrids

Ruba Almasri ^1,2^, Paul Joyce ^1,2^, Hayley B. Schultz ^1,2^, Nicky Thomas ^1,2^, Kristen E. Bremmell ^1,2^ and Clive A. Prestidge ^1,2^*

^1^ University of South Australia, UniSA Clinical and Health Sciences, Adelaide, South Australia 5000, Australia; Ruba.Almasri@mymail.unisa.edu.au (R.A.); Paul.Joyce@unisa.edu.au (P.J.); Hayley.Schultz@unisa.edu.au (H.S); Nicky.Thomas@unisa.edu.au (N.T.); Kristen.Bremmell@unisa.edu.au (K.B.);

^2^ ARC Centre of Excellence in Convergent Bio-Nano Science and Technology, University of South Australia, Adelaide, South Australia 5000, Australia

***** Clive.Prestidge@unisa.edu.au (C.P.); Tel.: +61 8 830 22438

**Table S1.** Drug loading efficiencies of 3 FEN-loaded formulations (values represent mean ± SD, n=3).

| **Formulation** | **Drug Load**  **%(w/w)** | **Efficiency (%w/w)** |
| --- | --- | --- |
| 80% FS C300 | 2.7 ± 0.3 | 94 ± 2.8 |
| 200% FS PG8 | 16.7 ± 0.99 | 98.3 ± 4.5 |
| 400% MPS PG8 | 14.9 ± 0.6 | 94.4 ± 3.9 |

**Table S2.** The lipid content of the formulations and lipid dosed to lipolysis vessel.

| **Formulation** | **Drug dosed (mg)** | **Formulation dosed (mg)** | **Lipid**  **(% w/w)** | **Lipid dosed**  **(mg)** | **Lipid ratio** |
| --- | --- | --- | --- | --- | --- |
| 80% Liquid PG8 | 3 | 41.1 | 93 | 38.2 | 0.78 |
| 80% FS PG8  80% MPS PG8 | 3 | 79.5 | 48.1 | 38.2 | 0.78 |
| 200% FS PG8  200% MPS PG8 | 3 | 33.6 | 45.5 | 15.3 | 0.31 |
| 80% Liquid-C300 | 3 | 51.7 | 94.2 | 48.7 | 1 |
| 80% FS C300  80% MPS C300 | 3 | 100.5 | 48.5 | 48.7 | 1 |
| 200% FS C300  200% MPS C300 | 3 | 42 | 46.4 | 19.5 | 0.4 |

**Figure S1.** The relationship between XRPD peak intensity at 22.3° (2θ) and supersaturated drug loading (S_eq_) for SDA PG8 formulations and P PG8 formulations.

**Figure S2.** The % phase partitioning of FEN between the aqueous phase and pellet over 90 min after a 3 mg FEN dose of 80% formulations, Crystalline FEN and APO-fenofibrate, under biorelevant gastric and intestinal conditions. Values represent mean ± SD, n=3.

**Figure S3.** The % phase partitioning of FEN between the aqueous phase and pellet over 90 min after a 3 mg FEN dose of supersaturated formulations, under biorelevant gastric and intestinal conditions. Values represent mean ± SD, n=3.
